# A complete genome of an obligately lytic *Pseudomonas aeruginosa* bacteriophage, Minga-mokiny 4

**DOI:** 10.1128/mra.01289-24

**Published:** 2025-03-20

**Authors:** Phoebe G. Carr, Kak-Ming Ling, Joshua J. Iszatt, Wee Peng Poh, Erika. N. Sutanto, Renee N. Ng, Barbara J. Chang, Stephen M. Stick, Anthony Kicic, WAERP, AREST CF

**Affiliations:** 1 Wal-Yan Respiratory Research Centre, The Kids Research Institute Australia, The University of Western Australia 2720 , Perth, Western Australia, Australia; 2 Occupation, Environment and Safety, School of Population Health, Curtin University 64827 , Perth, Western Australia, Australia; 3 School of Biomedical Sciences, University of Western Australia 2720 , Perth, Western Australia, Australia; 4 The Marshall Centre for Infectious Diseases Research and Training, School of Biomedical Sciences, The University of Western Australia 2720 , Perth, Western Australia, Australia; 5 Department of Respiratory and Sleep Medicine, Perth Children's Hospital 60081 , Nedlands, Western Australia, Australia; 6 Centre for Cell Therapy and Regenerative Medicine, School of Medicine and Pharmacology, The University of Western Australia and Harry Perkins Institute of Medical Research 172098 , Perth, Western Australia, Australia; 7 St. John of God Hospital 60135 , Subiaco, Perth, Western Australia, Australia; 8 Murdoch Children’s Research Institute 559513 , Melbourne, Victoria, Australia; 9 Department of Pediatrics, University of Melbourne 2281 , Melbourne, Victoria, Australia; Department of Biology, Queens College, Queens, New York, USA

**Keywords:** phage, bacteriophages, *Pseudomonas aeruginosa*, genomes

## Abstract

We report the isolation of a bacteriophage with obligately lytic activity against *Pseudomonas aeruginosa* from wastewater. The reported phage, Minga-mokiny 4, appears to belong to the *Schitoviridae* family, is of the *Litunavirus* genus, and has a 72,362-bp genome. No known genes associated with lysogeny, bacterial resistance, or virulence were predicted.

## ANNOUNCEMENT

Infection with *Pseudomonas aeruginosa*, a Gram-negative and environmentally abundant bacterium, greatly impacts individuals who are immunocompromised or have chronic diseases. Conventional antibiotics often fail to effectively eradicate these infections, leading to the emergence of antimicrobial resistance (AMR) (1). The lack of investment in new antibiotics poses a public health threat in managing difficult-to-treat infections (2). Phage therapy offers promising biological treatment alternatives (3, 4). Here, we report the complete genomic sequence of the phage Minga-Mokiny 4, isolated from wastewater and specific for *P. aeruginosa*.

The wastewater sample was collected from a wastewater treatment plant in Perth, Australia (31.955839 S 115.793828 E) and filtered through a 0.22 µm filter. Minga-mokiny 4 was isolated by enriching 1:1 volume of wastewater with Luria–Bertani broth at 37°C within an orbital shaker at 50  rpm and incubated overnight with its propagating host, *P.aeruginosa* isolate M1C90 (isolated from a child with cystic fibrosis [CF]; AREST CF, Melbourne, Australia) (5). The double-overlay method was used for isolation, followed by three rounds of plaque purification (6). Purified phage suspensions were then treated with DNase I and RNase A to eliminate bacterial genome contamination, while proteinase K treatment was performed to destroy phage capsid, using a Qiagen DNeasy Blood and Tissue Kit following the manufacturer’s protocol (7). Library preparation was conducted using a Nextera XT preparation kit and sequenced by the Australian Genomic Research Facility (Victoria, Australia) using the Illumina NovaSeq 6000 (Illumina, USA) platform, generating 3,133,596 150 bp paired-end (PE) raw reads.

Assembly steps and putative phage contig identification were conducted using the Phanatic v2.2.4 pipeline (8). Briefly, Phanatic conducted adaptor trimming and deduplication using BBTools v38.18 (9), followed by genome assembly using SPAdes v3.15.4 (10). Phanatic also calculated basic read statistics using BBTools v38.18 and assessed genome completeness using CheckV v1.0.1 and the CheckV database v1.5 (11). The genome was screened for bacterial resistance and virulence genes using ABRicate v1.0.0 (12), with default databases. The genome was annotated by Prokka v1.14.6 (13) using the Prokaryotic Virus Remote Homologous Groups (PHROGS) database and reordered based on the small terminase subunit (14). Phage taxonomy was classified with vConTACT2 (15) and taxmyPHAGE (16). The closest relative genomes to Minga-mokiny 4 were identified with the Basic Local Alignment Search Tool (BLASTn) (17) using the National Center for Biotechnology Information (NCBI) nucleotide database (accessed on 20 June 2024). FastANI v1.34 (18) was used to calculate the average nucleotide identity (ANI) by conducting a pairwise genome comparison with the identified closest genomes. Unless otherwise specified, default configurations and parameters were used for all software.

The characteristics of Minga-mokiny 4 are summarized in Table 1. The genome was classified as “complete” with a high-level confidence, and no lysogeny, bacterial resistance, or virulence genes were predicted. Minga-mokiny 4 exhibited a > 95% ANI score with its three closest relative sequences. Following published guidelines for demarcation of taxonomic ranks, using a consensus of taxonomic classification data, we can infer that this phage belongs to the genus *Litunavirus* (19). These reported characteristics make Minga-mokiny 4 a potential therapeutic candidate; however, further investigation is warranted.

**TABLE 1:** Characteristics of Minga-mokiny 4 and comparative analysis of its three closest relatives

Genome	Genome size (bp)	GC content (%)	No. of CDS	No. of tRNAs	Normalized sequencing depth (x)	CheckV completeness (%)	Closest relative phage (Genbank accession no.)	Average nucleotide identity (%)
Minga-mokiny 4	72,362	54.79	90	0	357	100	OM937766.1	95.88
							OM234792.1	96.52
							NC_070864.1	96.34

## Data Availability

The complete sequence of Minga-Mokiny 4 has been deposited in GenBank under the accession number PQ261042, BioSample number SAMN43359102, BioProject number PRJNA862682, and raw reads are available in the Sequence Read Archive under number SRR30420680
